# Second-Generation Bioethanol from Coconut Husk

**DOI:** 10.1155/2018/4916497

**Published:** 2018-09-27

**Authors:** Maria Bolivar-Telleria, Cárita Turbay, Luiza Favarato, Tarcio Carneiro, Ronaldo S. de Biasi, A. Alberto R. Fernandes, Alexandre M. C. Santos, Patricia M. B. Fernandes

**Affiliations:** ^1^Biotechnology Core, Federal University of Espírito Santo, Vitória, ES, Brazil; ^2^Military Institute of Engineering, Rio de Janeiro, RJ, Brazil

## Abstract

Coconut palm (*Cocos nucifera*) is an important commercial crop in many tropical countries, but its industry generates large amounts of residue. One way to address this problem is to use this residue, coconut husk, to produce second-generation (2G) ethanol. The aim of this review is to describe the methods that have been used to produce bioethanol from coconut husk and to suggest ways to improve different steps of the process. The analysis performed in this review determined that alkaline pretreatment is the best choice for its delignification potential. It was also observed that although most reported studies use enzymes to perform hydrolysis, acid hydrolysis is a good alternative. Finally, ethanol production using different microorganisms and fermentation strategies is discussed and the possibility of obtaining other added-value products from coconut husk components by using a biorefinery scheme is addressed.

## 1. Introduction

Modern life demands high mobility and, as a result, transport is one of the largest and fastest growing energy demanding sectors [1]. Also, increase in competitive agribusiness automatization leads to a high energy demand [2]. However, due to concern on the negative impact of fossil fuels on the environment, the use of biofuels emerges as a promising alternative that is gradually becoming technically and economically feasible [3].

Modern ethanol industry began in the 1970s when petroleum-based fuel became expensive and environmental concerns arose. In 1975, the Brazilian government launched a pioneer program known as “Proálcool” (Pro-Alcohol) with two main objectives: to reduce the impact caused by oil price increases and, at the same time, mitigate the fall of sugar price in the international market [4, 5]. Between 1980 and 2002, over five billion dollars were invested on sugarcane agriculture and industry to expand alcohol fuel production [5]. “Proálcool” is known worldwide for its positive effect on biofuel promotion [6]. Nowadays, in Brazil, 20 to 25% of anhydrous ethanol is used as an additive in gasoline. Moreover, since 2003, flex-fuel vehicles, which can use alcohol, gasoline or gasoline+alcohol, are on the market [5].

Ethanol is the leading liquid biofuel used for transportation. First-generation ethanol has a simple production process using sugar or grain as raw material (sugarcane juice in Brazil and corn in the USA and EU, for example), while 2G ethanol (bioethanol) has more complex steps of production and uses lignocellulosic material as a substrate [7]. Among the major byproducts generated by agroindustries, lignocellulosic biomass is one of the most abundant, conflict-free with food production and is available throughout the year at low prices [8, 9]. All of these characteristics show that lignocellulose waste might be considered the most feasible option for fossil fuel replacement, having a significant potential for bioethanol productivity while giving a destination for an environmental liability.

In Brazil, GranBio and Raízen are pioneering companies that utilize sugarcane coproducts as a substrate, enhancing ethanol production without increasing the cultivated area [10, 11]. In 2003, Raízen was able to produce more than 40 million liters per year [11]. In the USA, three companies produce cellulosic ethanol in commercial scale: POET-DSM Advanced Biofuels, DuPont, and ABENGOA [12]. There are many projects around the world focusing on the use of lignocellulosic residues for biofuel production [13]. These residues can come from homes or city dumps: companies in Canada are investing in the construction and operation of a renewable fuel plant using local residential kitchen and yard waste; Phuket's Provincial Administration Organization, in Thailand, is building a waste-to-biofuel facility that will use the municipal solid waste of the entire island as feedstock [14]. China's State Development & Investment Corporation began the construction of its first ethanol plant in the Liaoning province with 300,000 tons capacity and is planning to build five ethanol plants in other provinces [15]. Nowadays, biofuels have an important part in the global liquid fuel market and over a hundred companies in different countries base their production on various types of 2G biofuels [13]. Coconut husk is a very promising substrate that can be used as raw material for 2G ethanol production, since coconut palm plays an important role in the economy of several tropical countries [16]. The food industry uses coconuts to obtain various products leaving the husk as waste. It is important to note that coconut husk has a high lignin content that during husk decomposition penetrates the soil and can reach the water table imposing a great environmental risk. Since it is discarded in high volumes (coconut husk encompasses 80 to 85% of the weight of the fruit [17, 18], while sugarcane bagasse corresponds only to 27 to 28% dry weight), it is mandatory to find a safe destination for this waste. Therefore, the use of coconut husk for 2G ethanol production may be a solution to reduce the environmental impact. Moreover, if the technology is cheap and simple enough it can be used by small producers.

The three main components of a biomass (cellulose, hemicellulose, and lignin) form a recalcitrant structure, making it difficult for enzymes to have complete access to cellulose for conversion to monosaccharides. To make this feasible, the first major step in bioethanol production is biomass pretreatment (biological, physical or chemical), in which the lignin content is reduced to release the fermentable sugars from the rigid structure and, therefore, prepare the biomass for enzymatic conversion [19]. Different types of biomasses have different amounts and types of sugars (hemicellulose and cellulose) and lignin, so knowing its composition is crucial for the process. Moreover, the abundance of the residue must be taken into account so that the whole process is economically feasible.

The second major step in 2G ethanol production is the hydrolysis that unlocks and saccharifies the polysaccharides that are present in the biomass to fermentable sugars [20]. Generally, enzyme cocktails are used to catalyze reactions to obtain simple sugars such as glucose and mannose for further fermentation by microorganisms. This process, also called saccharification, is very important and the requirements of these enzyme complexes, which act synergistically, add major costs to the overall process. The main challenge is to obtain a cost-effective technology of enzymatic hydrolysis for economically viable biofuels [20].

The fermentation process is the next step, in which a microorganism such as the yeast* Saccharomyces cerevisiae* ferments the sugars that are present in the treated biomass and produces ethanol [21]. To increase the economic feasibility of this process, industries show great interest in using yeast strains that are more tolerant and resistant to various kinds of stresses and that are also able to use pentoses that come from hemicellulose degradation, such as xylose, as most strains naturally only consume hexoses.

Currently, the process for 2G bioethanol production in large scale is being improved, since it still has cost production issues that derive from the procedures needed to overcome the recalcitrance of the lignocellulose (pretreatment and enzymatic hydrolysis) in order to obtain fermentable sugars [22]. To transform the bioethanol production into a sustainable and economically viable process, it is important to integrate it in a biorefinery, which is a great supporter of a biobased economy. In a biorefinery, almost all types of biomass residues can be converted to different classes of biofuels, biomaterials and other marketable bioproducts through jointly applied conversion technologies [23].

Although many articles address the use of coconut husk as a raw material for bioethanol production, few of them compare the results obtained using different methods. This work intends to give an overview on different approaches already tested to obtain ethanol from coconut husk to facilitate the development of a process that can effectively produce ethanol from coconut residue.

## 2. The Coconut Plant and Industry

Coconut palm tree is a perennial crop grown in tropical climate countries which present ideal conditions for its cultivation, such as soil with proper water capacity and drainage and warm ambient temperatures [24, 25]. Due to the coconut structure, many valuable products can be obtained from it, such as meat (copra), oil, water, milk, and fibers [25]; therefore, this fruit is of great economic importance. There are two major varieties, the tall (Typica), mainly used to obtain coconut meat and milk, and the dwarf (Nana), the most cultivated in Brazil, used for coconut water extraction [24, 25].

Coconut harvesting time is determined by its purpose and is usually carried out in two stages of ripening. The green fruits are destined to the coconut water market, while mature fruits are destined to the dry coconut market (for meat, milk and oil) [26]. Therefore, depending on the plantation site, the residue is made of green or mature coconut husks, which have different compositions (Table 1).

**Figure 1:**
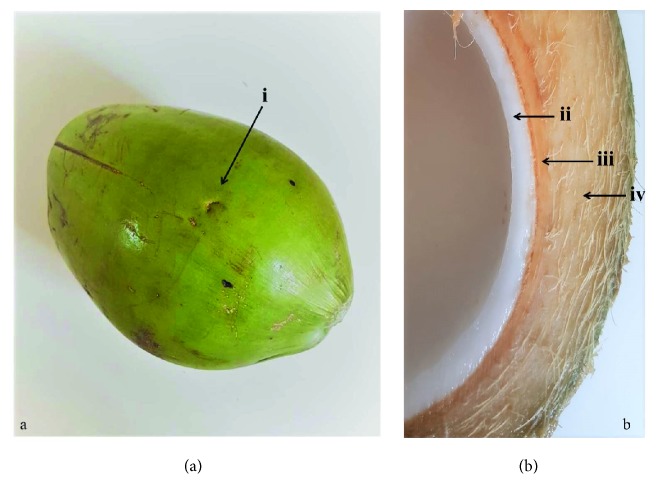
Green coconut and its structures. (a) Green coconut. (b) Green coconut without epicarp and liquid albumen. i, epicarp; ii, mesocarp; iii, endocarp; iv, solid albumen.

The coconut fruit has a smooth green epidermis (epicarp), a medium region with bundles of fibers (mesocarp), and a stony layer that surrounds its edible part (endocarp) (Figure 1).

The estimated annual worldwide coconut production in 2015 was around 55 million tons and the main producing countries are Brazil, India, Indonesia, the Philippines, and Sri Lanka [16, 17]. Indonesia is responsible for 33.1% of the total world production [24], and the coconut industry plays a significant economic role in this country as well as in other tropical countries. However, as mentioned before, 80 to 85% of the weight of the fruit is not used and is simply discarded, resulting in large amount of waste [17, 18]. Also, the coconut husk is rich in phenolic compounds, which are toxic to humans and animals and are released in the environment as a result of natural deterioration [39]. Actually, only a small percentage of the total fiber residue is designated for the production of fertilizers and handmade products like mats, nets, and brooms [17, 40], and, unfortunately, the traditional process to obtain the coir fiber is highly polluting as it is performed in surface waters and, again, liberates polyphenols [17, 40]. Due to high residue volumes and the husk slow decomposition, the coconut industry turns into an environmental and handling problem [41]. A possible and sustainable solution for the coconut husk residue is the production of 2G bioethanol.

Currently, around 20 published scientific papers describe the study of coconut husk as raw material for bioethanol. This means that it is an interesting opportunity to contribute to this field of study, allowing, hopefully in a near future, that small and local producers to produce biofuels for their personal use and for the development of a sustainable coconut chain production. As mentioned earlier, coconut producing countries are part of the third-world economy; in this way, turning trash into jobs and income generation is a very important matter.

## 3. Methods Used in Coconut Conversion to Ethanol

### 3.1. Coconut Husk Pretreatment for Bioethanol Production

The three main components of lignocellulosic biomass, cellulose, hemicellulose, and lignin, form a strong matrix, which gives it recalcitrance, meaning low enzyme digestibility [42]. Biomass used for bioethanol production has to undergo a pretreatment to remove lignin and hemicellulose and overcome recalcitrance by increasing porosity and reducing cellulose crystallinity making it available for biological or chemical hydrolysis. An effective pretreatment must return high sugar concentration but always avoiding their loss and degradation; also it has to minimize formation of inhibitors and be able to undergo fermentation without detoxification, to reduce process steps, water, and energy consumption in order to decrease costs [43–45]. The pretreatment is a very important step as it has an impact on the next stages, such as hydrolysis, fermentation, and downstream processing [43]. Since biomass composition varies from one substrate to another, different pretreatments have to be tested to find the best for each specific substrate.

The first step in the pretreatment is the substrate preparation to make the enzymatic hydrolysis more effective by mechanically reducing cellulose crystallinity [45]. In the case of coconut husk, it is dried, ground, and sieved to obtain a powder [31].

The most used pretreatment for coconut husk is alkaline, followed by acid, but there are also other methods being tested that will be discussed in this section (Table 2).

Depending on biomass composition and the type and conditions of the pretreatment, products that inhibit enzymatic hydrolysis and fermentation, such as weak acids, furfural, 5-hydroxymethyl furfural (HMF) and phenolic compounds are formed [42]. After the pretreatment, most authors wash the pretreated coconut husk to extract inhibitors from the biomass [27–29, 46, 47]. This approach might not be the best as it increases the number of process steps and uses more water which affect the cost, and, moreover, a high content of sugars are lost during these washes [29, 48].

#### 3.1.1. Alkaline Pretreatment

The main effect of alkaline pretreatment is delignification of the biomass and reduction of crystallinity [29, 43–45]. For these pretreatments, sodium, potassium, calcium, and ammonium hydroxides and ammonia are used [44, 45]. All revised studies that used alkaline pretreatment in coconut husk used NaOH and most of them use high temperatures [27–29, 33, 46, 48–50].

Soares et al. [50] proposed a pretreatment with dilute NaOH (1% (w/v)) at room temperature to decrease the formation of inhibitors, using high-solid loadings (18% (w/v)) and no detoxification of the pretreated biomass to obtain higher sugar concentration. In a later work, Soares et al. [48] suggest the use of a fed-batch pretreatment and saccharification with higher solid loadings (25 and 30% (w/v)).

#### 3.1.2. Acid Pretreatment

Acid pretreatment is a widely used and effective method for obtaining high yields of sugars from lignocellulosic biomass. Fatmawati and Agustriyanto [47] and da Costa Nogueira et al. [29] pretreated coconut husks with diluted acid (1.5% and 3% (w/v) of H_2_SO_4_) and autoclaved at 121°C. De Araújo et al. [51] tried an acid pretreatment followed by an alkaline treatment of the washed neutralized fibers, both at high temperature.

#### 3.1.3. Other Pretreatment Approaches

Pretreatments using alkaline conditions combined with other techniques have been tested for coconut husk. One approach consists of presoaking the coconut husks in a NaOH solution and then microwaving them [52, 53]. Other pretreatments use a combination of alkaline and oxidative conditions [31, 51], where an H_2_O_2_ solution is adjusted to pH 11.5 with NaOH.

Gonçalves et al. [30] used a two-step method to remove different components from the husk. First, they utilized oxidative conditions (NaClO_2_–C_2_H_4_O_2_) to remove the lignin. Then, they performed autohydrolysis to extract the hemicellulose.

Other pretreatments reported for coconut husk are the use of high temperature for autohydrolysis [29, 32, 53], use of aqueous glycerol and acidified aqueous glycerol at 130°C [54], and use of the surfactant Tween® 80 during acid, alkaline, and hydrothermal pretreatment to increase enzymatic hydrolysis [29].

### 3.2. Hydrolysis

The next step in bioethanol production is breaking cellulose and hemicellulose into simple sugar monomers that can be fermented. Cellulose is hydrolyzed to glucose, while hemicellulose hydrolysis releases a mixture of pentoses and hexoses [44]. There are different hydrolysis strategies like dilute and concentrated acid, alkaline, hot-compressed water, and enzymatic [55]. Enzymatic hydrolysis is the most widely used as it is the most ecofriendly, has no formation of inhibitors, requires less energy, and is operated at mild conditions (40-50°C and pH 4-5) so there are no corrosion problems [20, 44, 56]. On the other hand, alkaline and acid hydrolysis present high toxicity, high utility cost, lower sugar yields, and corrosion, along with the formation of inhibitors [44, 56].

**Figure 2:**
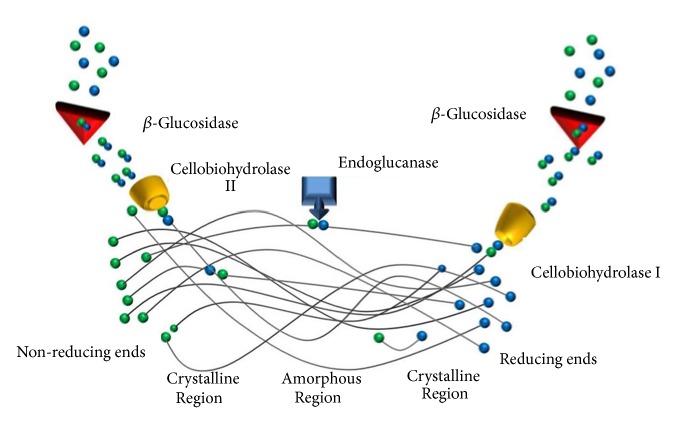
Hydrolysis mechanism of cellulose by cellulase cocktail components. Endoglucanases cleave the inner region of cellulose, the reducing and non-reducing regions are hydrolyzed by cellobiohydrolases (I and II) and the cellobiose is hydrolyzed to glucoses by *β*-glucosidase [34]. Adapted from Wang et al. [35].

A cocktail of enzymes composed of cellulases and hemicellulases that work in synergy is needed to effectively hydrolyze the cellulose and hemicellulose (Figure 2) [34, 44, 56]. These enzymes are naturally produced by various fungi and bacteria. The fungus* Trichoderma reesei* is one the most used industrially to produce cellulases [57]. These strains have been engineered to produce a large amount of chosen cellulases (native, homologous, or engineered) so they have a high specific activity on crystalline cellulose [58, 59]. Currently, the most advanced cocktails in the market are Cellic® CTec3 from Novozymes (Bagsværd, Denmark) and Accellerase® TRIO™ from DuPont Genencor (CA, USA) [58].

Enzymatic hydrolysis is the economic bottleneck of lignocellulosic bioethanol production because of its high cost. Enzyme production costs comprise 25 to 50% of bioethanol production cost [60]. Efforts have been made to decrease enzyme price and it has dropped from US$ 5 per gallon or US$ 0.75 per liter of bioethanol to US$ 0.10–0.18 per gallon or US$ 0.027 per liter of bioethanol [34].

Producing better enzymes with higher efficiency using lower doses are important to make lignocellulosic bioethanol economically feasible. There are many factors that affect the efficiency of enzymatic hydrolysis including temperature, pH, mixing rate, enzyme loading, pretreatment, present inhibitors, substrate type and concentration (can lead to inhibition), and end-product inhibition (glucose) [44, 56]. As a result, the development of enzymes with (i) stability at higher temperatures and pH, (ii) increased tolerance to pretreatment inhibitors and end-product inhibition, (iii) better efficiency, (iv) higher adsorption, and (v) catalytic efficiency is highly needed [20]. For example, new thermophilic strains such as* M. thermophila* C1 are used to produce cellulases with broader pH and temperature ranges that also contain richer hemicellulases [58].

In the case of bioethanol production from coconut husk, most studies use enzymatic hydrolysis, where the use of commercial cocktails is the most common approach [27, 29, 31–33, 46–54]. Another method is to isolate fungi from the substrate to be used for bioethanol production hoping to find a microorganism with high specificity for that biomass as was done by Albuquerque et al. [46] for fresh and rotting coconut husk. The best isolates (*Penicillium variable* and* Trichoderma* sp.) were used to produce enzymes by submerged fermentation.

Some studies have been made to test the use of surfactants to improve enzymatic hydrolysis in coconut husk but using different approaches. Da Costa Nogueira et al. [29] used Tween® 80 during different pretreatments, while de Araújo et al. [51] used rhamnolipids produced by* Pseudomonas aeruginosa* during enzymatic hydrolysis. Rhamnolipids are biosurfactants that, unlike chemical surfactants, are biodegradable and that makes them an environmentally friendly option.

Non-enzymatic hydrolysis methods have also been tested for coconut husk. Acid hydrolysis using sulfuric acid 1, 2, 3, and 4% (v/v) and high temperature after an alkaline pretreatment was performed by Jannah and Asip [28]. Moreover, Prado et al. [61, 62] employed subcritical water hydrolysis, which utilizes pressure to maintain the water in a liquid state using coconut husk without any pretreatment.

### 3.3. Fermentation

Microbial fermentation is the next step in the production of lignocellulosic bioethanol in which the fermentable sugars, such as glucose and mannose, obtained in the saccharification, are converted to ethanol [63].

*Saccharomyces cerevisiae* has traditionally been used to produce alcohol in brewing and wine industries [21]. This yeast produces high yields of ethanol with high productivity [64, 65]. Nowadays, other yeasts and bacteria are also used for bioethanol production [9]. Using other microorganisms with different characteristics from* S. cerevisiae* or a combination of microorganisms (cofermentation) can increase ethanol yield. For example, using yeasts that naturally ferment pentoses, such as the xylose consuming* Pichia (Scheffersomyces) stipitis*,* Candida shehatae* and* Pachysolen tannophilus *[66], or bacteria such as* Zymomonas mobilis, *which presents fermentation under anaerobic conditions, high ethanol tolerance, and high ethanol-producing capacity [67], can increase the final ethanol concentration.

Another approach to overcome the challenges in lignocellulosic bioethanol production is the development of genetically engineered microorganisms that are capable of fermenting pentoses and hexoses. Engineered yeast strains with these characteristics are more economically viable for industrial production of bioethanol [68]. With development of new DNA editing technology, the metabolic potentials of microorganisms are being explored and harnessed in plenty new ways. The development of strains that can ferment xylose, the main pentose in coconut husk, is done by inserting genes related to the degradation pathway of this pentose, like overexpressing genes related to the pentose phosphate pathway (such as TKL1 and TAL1) [69, 70]. Other strategies are decreasing the formation of xylitol as it is a harmful derivative for the complete fermentation of the pentoses and preventing the ubiquitination of hexose transporters, since they also act to carry pentoses, but suffer degradation when the concentration (or absence) of glucose decreases [71, 72]. Ethanol production can also be improved by preventing the formation of glycerol, another fermentation product. It has been shown that the deletion of genes in this pathway like GPD2 and FPS1 is related to an improvement in the final ethanol production, since they redirect the metabolic flow to the alcoholic fermentation [73, 74]. Another approach is the interruption of the ADH2 gene, related to the transformation of ethanol into aldehyde, as it has higher affinity for ethanol compared to other isoenzymes [75]. Genetic engineering can also be used to obtain microorganisms that are more tolerant to stresses like inhibitors produced during the pretreatment and a high ethanol concentration that is present at the end of the process. This can be done by inserting genes, such as* Saccharomycopsis fibuligera *TPS1 (6-phosphate-trehalose synthase) into* Saccharomyces cerevisiae*, or fine-tuned proteins such as RNA pol2 responsible for mRNA expression [76, 77]. Genetic engineering offers the advantage over traditional methods of increasing molecular diversity in a direct, specific, and faster way.

### 3.4. Hydrolysis and Fermentation Strategies

There are three main strategies for hydrolysis and fermentation: separate hydrolysis and fermentation (SHF), simultaneous hydrolysis and fermentation (SSF), and semi-simultaneous hydrolysis and fermentation (SSSF). In SHF the hydrolysis is done at a higher temperature which is optimal for the enzymes and later the fermentation is performed at a lower temperature optimal for the microorganism. On the other hand, in SSF the enzymatic hydrolysis and the fermentation are executed at the same time at an intermediate temperature. This strategy helps to reduce processing times, sugar inhibition, and equipment cost, since only one vessel is needed [78]. The major problem is that the temperature is not optimal for the enzymes and sometimes for the microorganisms (the use of microorganisms with higher optimal temperature solves this last problem).

A way to obtain the advantages of both SHF and SSF is to include a prehydrolysis step before inoculation, which is performed at an optimal temperature for the enzyme, followed by an SSF. This method is called SSSF. Some of the advantages of using SSSF are no carbon deficiency in early stages as presented during SSF [78], higher enzymatic activity during prehydrolysis because of optimal enzyme temperature, and reduction of slurry viscosity, which enables higher solid loadings and easier stirring and pumping [79].

## 4. Results and Discussion

### 4.1. Coconut Husk Pretreatment for Bioethanol Production

A strategy to evaluate the effectiveness of a pretreatment is to compare the composition of the biomass before and after the procedure. This is a key parameter to know whether the technique removes the lignin, degrades the hemicellulose, and conserves the cellulose. However, since the sugar concentration after hydrolysis depends on many factors, the biomass with the most changes will not yield necessary the highest sugar turnout. Therefore, both the coconut husk composition after pretreatment and the sugar concentration after hydrolysis should be taken into account in any study.

Alkaline pretreatment seems to be the best approach to obtain sugars from coconut husk probably because it helps to remove the lignin from the substrate. The results of delignification with NaOH are reported by Gonçalves et al. [33] and Jannah and Asip [28]. As for hemicellulose content, two studies show an increase [27, 29], while other two show a decrease [33, 49]. This is probably due to the conditions used on each work. The highest increase in cellulose content was observed by Gonçalves et al. [33] and Cabral et al. [49]. Of the studies with composition analysis after pretreatment, Gonçalves et al. [33] obtained the best results using NaOH pretreatment, with high cellulose increase and high delignification. On the other hand, Vaithanomsat et al. [27] and da Costa Nogueira et al. [29] observed only a small increase in cellulose content and delignification in comparison with other methods.

Also, the studies that presented highest sugar concentrations after hydrolysis used alkaline pretreatment with NaOH (the results will be discussed in the hydrolysis section) [27–29, 48, 50]. It was observed that higher NaOH concentrations, temperature, and processing time produce more inhibitors, which may affect the next steps of the process [33, 48].

Soares et al. [50] selected mild alkaline conditions (1% NaOH (w/v), room temperature, and shorter reaction time) to decrease the formation of inhibitors. They also proposed no detoxification of the pretreated biomass and the use of high-solid loadings (18% (w/v)), which improved sugar release over most of the other studies, and consequently ethanol concentration. In a later study, Soares et al. [48] used the same mild conditions but did a fed-batch pretreatment and saccharification increasing the solid loadings to 25 and 30% (w/v), which increased the final sugar and ethanol concentration. However, rising the solids loading up to 30% also led to a diminution in the yield (g ethanol/g sugar) but also showed one of highest sugar concentrations.

The use of high-solid loadings (≥ 15% solids, (w/v)) during the pretreatment and/or hydrolysis stages brings economic benefits such as less energy consumption during the processes, including distillation, and use of smaller vessels and equipment, which translates to lower capital cost [80]. Unfortunately, it also implies many setbacks, including a higher concentration of inhibitors, mass transfer limitations and reduction of ethanol yield as solid loadings rise [80, 81]. As solid loadings increase, free water decreases and viscosity rises; as a result, there is a reduction in the effectiveness of the pretreatment and enzymatic efficiency because of poor diffusion and solubilization [80–83]. High viscosity also brings handling problems, as mixing, pumping, and pouring become harder [82]. There are different approaches to reduce viscosity such as the use of surfactants [84] and employing a fed-batch process, which unfortunately shows a decline in conversion when more solids are introduced [81] as observed by Soares et al. [48].

Da Costa Nogueira et al. [29] compared alkaline, acid, and autohydrolysis pretreatments and the alkaline pretreatment showed the highest final sugars concentration. The composition of the husk was almost unchanged by the autohydrolysis pretreatment and the composition after acid pretreatment was not shown. They also showed that adding Tween® 80 during alkaline pretreatment can increase final sugars concentration by obtaining a higher digestibility during the enzymatic hydrolysis, but no difference was seen when acid and autohydrolysis pretreatments were performed with or without the surfactant [29].

Ding et al. [53] showed best results for microwave-assisted-alkaline pretreatment, followed by alkaline, then acid and lastly autohydrolysis. Unfortunately, all pretreatments in this study led to a low sugars concentration. The delignification obtained by microwave-assisted-alkaline pretreatment was significant and led to a significant increase in cellulose and hemicellulose concentration. Other works that used autohydrolysis also obtained better results using other pretreatments [29, 32].

The studies that used H_2_O_2_ in alkaline conditions showed a large difference in the sugars concentration. This might be due to the delignification with NaOH performed by Gonçalves et al. [31] after the pretreatment, which resulted in higher delignification and increased cellulose and sugar concentration relative to the values reported by de Araújo et al. [51].

On the other hand, it was observed that dilute acid pretreatment is not the best strategy for obtaining sugars from green coconut fibers [29]. A possible explanation for dilute acid pretreatment not being the best strategy for obtaining sugars from green coconut fibers is that coconut husk has a high lignin content. Studies have shown that acidic pretreatment at high temperature forms lignin droplets that adhere to the biomass interfering with the enzymatic hydrolysis [85]. De Araújo et al. [51] reported no significant removal of lignin using acid pretreatment followed by an alkaline treatment at high temperature, which agrees with the low delignification reported by Fatmawati and Agustriyanto [47].

Another interesting pretreatment proposed by Gonçalves et al. [30] for coconut husk is the use of oxidative conditions (NaClO_2_- C_2_H_4_O_2_) for delignification and autohydrolysis for hemicellulose removal. The authors obtained a high sugar content after hydrolysis, a high delignification, and a reduction of hemicellulose as desired, but most lignin was conserved. In terms of lignin removal and cellulose increase, their results are similar to those of Gonçalves et al. [33] with NaOH at high temperature, but they also obtained a higher hemicellulose elimination. They also reported a higher difference in crystallinity than in other studies [29, 31–33].

### 4.2. Inhibitors of the Enzymatic Hydrolysis and Fermentation

The main inhibitors found in pretreated coconut husk are HFM, furfural, phenolic compounds, formic acid, and acetic acid, the last one in the highest concentrations (Table 2) [29, 33, 46, 48–50]. Soares et al. [50] showed a relationship between an increase in NaOH concentration in the pretreatment and the inhibitor concentration. After alkaline pretreatment, acetic and formic acids and phenolic compounds are the main inhibitors produced, but no HMF or furfural were detected [29, 50]. On the other hand, Gonçalves et al. [33] showed a rise of HMF, furfural and total phenolic compounds with increasing pH. These differences seen in the inhibitors found are due to differences in the pretreatment. Also, pretreated coconut husks that still have solid albumen present very high levels of fatty acids that act as strong inhibitors [48].

### 4.3. Hydrolysis

Table 3 presents a compilation of the results published so far. As it can be observed, each work uses a different approach, making it difficult to determine the best methodology for enzymatic hydrolysis. Different pretreatments (which affect the type and concentration of inhibitors), amount of total solid loadings, and type of enzymatic cocktail and doses are used in each study (Table 3). Nevertheless, it is expected that the enzymatic hydrolysis efficiency will be affected by the pretreatment method. It has been shown that mature coconut fiber has the highest enzymatic conversion yield after NaOH pretreatment at high temperatures (90.72%)[33], followed by autohydrolysis pretreatment (84.10%) [32] and, at last, the alkaline hydrogen peroxide pretreatment with a posterior NaOH delignification (76.21%) (Table 3)[31]. Interestingly, the highest glucose concentration was reported for the pretreatment with the lowest hydrolysis yield [31] and the lowest glucose concentration was determined for the pretreatment with the highest yield [33] (Table 3). This might be explained by a difference in initial cellulose composition for the enzymatic hydrolysis after the pretreatment and by the cellobiose concentration after hydrolysis, which is not reported.

There are also differences in the enzymatic performance depending on the severity of the pretreatment conditions. For example, coconut husk pretreated with 4% (w/v) NaOH gives lower sugar titers than with 1% (w/v) NaOH due to enzymatic inhibition. Also, the longer the pretreatment, the lowest the final sugar concentration [50].

Other factors might also influence enzymatic activity. As an example, Albuquerque et al. [46] used different hydrostatic pressures to improve the performance of fungi cellulases isolated from coconut husk in comparison to industrial cellulases. Actually, coconut fungi cellulases displayed better enzymatic activity on filter paper and on coconut husk hydrolysis than commercial cellulases at atmospheric pressure and at 300 MPa. These findings show that isolating native strains from the biomass can lead to highly specific cellulases, which lead to better results than commercial enzymes. They also demonstrated that high pressure can be used as pretreatment of cellulosic fibers as it promoted ruptures in the coconut fibers that helped in the later saccharification process. High hydrostatic pressure establishes interesting physical and consequently biological changes that can be used in biomass pretreatment and fermentation areas on biofuels synthesis and in the use of residual lignocellulosic materials with greater efficiency.

In the end, the main objective is to have the highest sugars concentration with the highest conversion yield possible. Some authors report very low total reducing sugars after hydrolysis of coconut husk like Fatmawati and Agustriyanto (1.2 g·L^−1^) [47] and Ding et al. (2.8 g·L^−1^) [53] (Table 3), which are far from the 8% (w/w) of glucose minimum required to make the distillation economical [81]. Nevertheless, three studies achieved over 8% (w/w) of sugars and all of them used alkaline pretreatment [28, 48, 50]. While two of the studies used enzymatic hydrolysis [48, 50], Jannah and Asip [28] performed an acid hydrolysis showing the highest sugars concentration with 4% (v/w) sulfuric acid but no information about inhibitors was reported (Table 3). Soares et al. [48] reached the highest sugars concentration using high-solids loadings in a fed-batch pretreatment and enzymatic saccharification, but they also observed a decrease of the conversion yield, which is characteristic of this kind of conditions.

As for the use of surfactants to enhance hydrolysis, da Costa Nogueira et al. [29] only obtained higher digestibility when using Tween® 80 during the alkaline pretreatment, whereas de Araújo et al. [51] found that adding rhamnolipids during hydrolysis improved cellulose conversion. Comparing both studies, da Costa Nogueira et al. [29] showed a higher hydrolysis yield in all conditions, presenting the best results for the coconut husk pretreated with NaOH and Tween® 80.

While using subcritical water hydrolysis, Prado et al. [61] obtained best results at 250°C and 20 MPa but observed an increase in the concentration of inhibitors (HMF and furfural) relative to processes at lower temperatures, where only hemicellulose is degraded. In this study, three different substrates were used and the results showed that coconut husk and palm fiber have similar final sugars concentrations (11.7 and 11.9%, respectively), but a defatted grape seed displays a much lower concentration (6.4%). On the other hand, using CO_2_ during the subcritical water hydrolysis resulted in a lower concentration of monosaccharides for coconut husk [62], which is detrimental for ethanol production. The advantages of subcritical water hydrolysis are the absence of polluting reagents, a reduction of process steps, no corrosion, less residue generation, and lower sugar degradation [61]. As a downside, high temperature and pressure are necessary.

Up to now, enzymatic hydrolysis has been the preferred method for coconut husk hydrolysis, but after reviewing all results it is evident that further investigation of the use of acid hydrolysis is necessary. An important factor that must be taken in account is the concentration of inhibitors after acid hydrolysis. It must be observed that the substances that are considered inhibitors of enzymatic hydrolysis may not be important in the case of acid hydrolysis if they do not affect the fermentation process. Measuring the concentration of other sugars would also be interesting to evaluate the real potential of acid hydrolysis compared to enzymatic hydrolysis. Other factors to take into account are the costs and the handling complexity of the process of acid hydrolysis, including corrosion due to acid conditions.

### 4.4. Fermentation

Results on which hydrolysis and fermentation approach is best for ethanol production differ as it is affected by many factors such as the enzymatic loading used, substrate, solid loadings, pretreatment, inhibitors, the microorganism used, and prehydrolysis time in SSSF [78].

Ebrahimi et al. [54] used a SSF approach and obtained a much higher ethanol concentration (similar to Gonçalves et al. [31]) when using acidified aqueous glycerol compared to the just aqueous glycerol (Table 4). Gonçalves et al. [31–33] compared SSF and SSSF for different pretreatments with coconut husk using three microorganisms. In all three studies, SSSF presented higher ethanol yield, final sugars concentration, and productivity (Table 4). They also proved that* S. cerevisiae, P. stipitis*, and* Z. mobilis* are suitable for fermenting the coconut husk hydrolysates showing similar sugar consumption patterns and kinetic parameters (ethanol yield, concentration, and productivity) for each separate pretreatment. For sequential alkaline hydrogen peroxide-sodium hydroxide [31] and autohydrolysis [32] pretreatments,* P. stipitis* showed slightly higher ethanol concentration and ethanol productivity than the other microorganisms but the ethanol yield was a bit lower. The highest ethanol concentrations (11-12 g·L^−1^), yield (84-92%), and productivity (0.23-0.32) for all strains were achieved with the NaOH pretreatment using high temperature with very little differences between microorganisms [33]. It is important to point out that the sugars concentration after hydrolysis reported on Table 3 for these three studies might not be of the same quantity as the one produced by SSF and SSSF as many interactions modify the enzymatic activity, so direct comparison of the ethanol produced with the glucose concentration obtained by hydrolysis is not recommended.

Soares et al. [50] compared ethanol production of coconut husk hydrolysate with two different* S. cerevisiae* strains, a commercial strain, Ethanol Red, and a genetically modified strain, GSE16-T18. This engineered strain can ferment xylose and resists fermentation inhibitors, leading to enhanced ethanol production (Table 4).

Once again, the largest concentrations of ethanol were obtained in processes that used alkaline pretreatment and used SFH [28, 48, 50], but only two [28, 48] are above the 4% (w/w), considered as the minimum ethanol concentration for an economically feasible production [81]. Soares et al. [48] obtained the highest sugars concentration using a fed-batch pretreatment and hydrolysis approach, but the ethanol concentration was smaller than that obtained by Jannah and Asip [28]. This is probably due to the fact that the sugars concentration reported by Jannah and Asip [28] is just glucose, while Soares et al. [48] used the total sugars concentration. This shows the importance of the way data are presented, since Jannah and Asip [28] are probably presenting less sugars than the ones that can be fermented and Soares et al. [48, 50] are showing a mix of sugars that may include some that are nonfermentable. The best way to report sugar concentration would be by showing separately the concentration of glucose, since it is the main sugar in the liquor, and that of total fermentable sugars (which varies depending on the microorganism used). Soares et al. [48, 50] determine ethanol yield based on the concentration of fermentable sugars, while Jannah and Asip [28] do not calculate the conversion yield. For coconut husk, all reported ethanol yields are based on the relationship between the mass of ethanol produced divided by the mass of fermentable sugars detected by HPLC, ignoring the ambiguity of using total reducing sugars as a parameter [27, 31–33, 50]. TRS is the easiest way to measure sugars but includes nonfermentable sugars, making it difficult to compare different processes and to evaluate the real efficiency of fermentation.

Vaithanomsat et al. [27] and Cabral et al. [49] showed an initial sugar concentration for fermentation higher than that reported after hydrolysis with no further explanation on how that increase occurred.

Currently, the use of genetically modified organisms and the use of microorganisms other than* Saccharomyces cerevisiae* or a mix of them (coculture) have been scarcely studied in the case of ethanol made from coconut husk. Since this biomass has a high hemicellulose content, the use of microorganisms that are able to ferment pentoses may help to increase the ethanol production.

### 4.5. Comparison with Other Biomasses

Bioethanol production conditions analyzed in studies similar to the present one (where various works about a biomass are compared), but for sugarcane bagasse [86, 87], wheat straw [88], and corn stover [89] were examined.

Zhao et al. [89] did a profound analysis of the literature for articles published during the last 10 years that used corn stover as a raw material for bioethanol production. By analyzing a high number of works on the subject (474), they were able to draw some conclusions that are hard to do when analyzing a much smaller sample and confirmed some of the observations obtained on this study. Regarding the pretreatment, they saw that two-thirds of the papers used acid, steam explosion, ammonia-based, and alkaline processes. In the beginning acid and steam explosion were the most popular but their use is declining, while solvent-based and combined techniques are gaining ground. Compared to coconut husk, which presented best results with alkaline pretreatment, corn stover showed highest ethanol production with alkaline, solvents, and ammonia pretreatment (19-22%) and lowest with fungi (11%). This low effectiveness with biological pretreatment was also reported for sugarcane bagasse [86].

The best results found by Cardona et al. [86] in 2010 for sugarcane bagasse were using acid hydrolysis (48% (w/w) TRS and 19 g etanol·L^−1^), but Bezerra and Ragauskas [87] in 2016 found the highest sugar and concentration with steam explosion (57.7 g glucose·L^−1^ and 25.6 g ethanol·L^−1^). Even though Cardona et al. [86] saw a higher glucose concentration when using alkaline pretreatment, they argue that costs are too high for the process to be viable at large scale. Talebnia et al. [88] reported steam explosion as the most suitable pretreatment for wheat straw because it has a lower reaction time, higher solid loadings, and a minimum use of chemicals. The best results for wheat straw were obtained with native non-adapted* S. cerevisiae *(31.2 g ethanol·kg^−1^, 99% ethanol yield).

As pointed out in this study for coconut husk, Zhao et al. [89] also remarks that most of the studies for corn stover are focused on the pretreatment. Fermentation is only reported in half of the studies and most use yeasts (92%) and the rest bacteria (8%). Also purification is usually not described and when it is they mostly use distillation.

Equal to the findings for coconut husk, enzymatic hydrolysis is used in most of the studies [86–89]. Zhao et al. [89] report that 95% of the articles for corn stover used enzymatic hydrolysis and, as also seen on this work, the enzyme doses differ significantly from study to study.

Parallel to the findings of this study, Zhao et al. [89] confirm that ethanol production varies greatly from one study to another even when using similar processes. For corn stover, ethanol conversion for most studies ranged between 80 and 100% with no significant difference while using different microorganisms for fermentation [89]. They also observed that xylose fermentation was a key factor for higher ethanol production, confirming the importance of not extracting the hemicellulose for fermentation and the need to use microorganisms that can ferment these sugars.

Most studies analyzed for this kind of technology are done in laboratory scale (98% for corn stover [89]), including coconut husk. Zhao et al. [89] observed that, in the case of corn stover, some pilot scale processes used smaller concentrations of chemicals than the concentration used in laboratory studies. No full scale plants for this kind of work are reported on the articles analyzed [86–89]. This is probably because it is not in the interest of industry to report its know-how and results.

### 4.6. Techno-Economic Overview on Bioethanol Production

Since there are no published data on the costs of bioethanol production from coconut husk, an extrapolation based on results from other biomasses was performed. Most of the techno-economic analyses on the production of biofuels were simulations for a few lignocellulosic feedstock, pretreatments, and enzymatic hydrolysis [19, 90–94]. Eggeman and Elander [90] made the economic analysis using different pretreatments for corn stover and found a similar minimum ethanol selling prices (MESPs) using dilute acid, hot water, ammonia fiber explosion (AFEX), ammonia recycle percolation (ARP), and lime pretreatments. Similar results were found by da Silva et al. [19] for hot water and AFEX pretreatment, but a higher MESP was obtained using dilute acid pretreatment. On the other hand, Chovau et al. [92] find dilute acid pretreatment as the best option.

The main factors that affect the MESP are plant size [91, 95], feedstock price and transportation [91, 93, 95–98], composition of the feedstock [91], pretreatment [94], enzyme cost and loading [91, 93, 96–98], conversion from cellulose to glucose [91, 93], ethanol yield [95], fermentation of pentoses [93], investment costs [93, 95], and energy cost [96–98]. Ethanol yield is relevant, since a higher yield means that less feedstock is required and overhead costs are smaller [95].

The MESP is also significantly affected by the location of the production, not only due to the availability, price, and transportation cost of the feedstock but because of the local technology, the cost of raw material (especially enzyme), the cost of energy, and the local policies. Zhao et al. [93] compared the MESP of a process using the technology available in China, which included using local enzymes with lower activity, so that higher loadings were needed and only hexoses were fermented, and technology from the United States of America using economic parameters from China. They observed that the MESP for their process with Chinese technology was above the local price of fuel ethanol, while the MESP obtained using more advanced technology was lower than the local price of fuel ethanol.

As observed by Chouvau et al. [92] the MESP reported by different authors varies greatly (from $0.21/L to $1.21/L) according to the assumptions involved. Most authors use a future expected cost for the enzymes that is much lower than the present cost and leads to a significant decrease of MESP [90–92]. Chovau et al. [92] observed an increase in reported MESP with higher enzyme cost when comparing various studies. They also estimated from these studies that about 13% of the MESP is due to enzyme cost. Some studies propose producing enzymes at the plant [92, 95], but Chovau et al. [92] reported higher costs for enzymes produced in-site due to energy consumption, higher investment, and lower plant capacity. As an alternative to enzyme use, acid hydrolysis, may be used, but recycling of acid is expensive and rises the costs [95].

Feedstock price also greatly affects the MESP, representing between 30 and 40% of it [92]. Corn stover price includes the grower payment, which is a compensation to the farmer for the fertilizers that he will use to recover the nutrients that would have been obtained from decomposition of the corn stover on the field [92]. This makes corn stover expensive and its price can vary greatly as fertilizer prices change annually and regionally. In addition, to reduce the transportation cost, which is significant, the plant has to be close to the source. Since feedstock price has a large impact on the MESP, it is important to use a realistic approach to this item when performing an economic simulation.

Macrelli et al. [97] studied the costs of using sugarcane to produce 1G and 2G (bagasse and leaves) in the same plant using steam pretreatment and enzymatic hydrolysis. By doing so, they achieved a lower MESP than when producing just 2G ethanol. This kind of scheme is already used by Raízen, as they produce sugar and later use the bagasse to produce 2G ethanol [11]. Therefore, they use the whole sugarcane and they do not have the extra transportation cost that most 2G ethanol plants have.

Duque et al. [98] and Quintero et al. [99] made an analysis using Aspen Plus™ for plants using agricultural residues from Colombia using acid pretreatment, enzymatic hydrolysis, and purification. Quintero et al. [99] added an energy generation facility powered mostly by lignin, while Duque et al. [98] did not include any heat exchange networks. Later Duque et al. [98] proposed to add such facility as their utilities represented 45.3% of the variable cost. Quintero et al. [99] also show the importance of including an energy generation system by comparing the MESP with and without this system. They obtained a lower MESP for all the biomasses when generating their own energy.

## 5. Future Perspectives

Bioethanol production from coconut husk might be a way to benefit rural development as it is mainly obtained by small producers. This way producers or cooperatives in rural areas might obtain a fuel for personal use enhancing energy security [1] and reducing waste volume, hence the environmental impact that the husks discarding brings. In order to make this possible it is crucial to have a simple and low-cost process by developing an appropriate pretreatment and access to cheaper enzymes. Since coconut husk is an agroindustrial residue, it should enhance competitiveness and social acceptance [8], as well as not presenting the ethical issue found when food crops are used to produce biofuels.

Producing bioethanol from coconut husk still has many challenges starting from the low concentration of sugars achieved in most of the studies, less than the 8% (w/w) of glucose needed to get a minimum of 4% (w/w) of ethanol to have an economically viable process [81]. After surpassing these sugar and ethanol concentrations the scale-up of the process must be done, where new challenges await, such as the decrease of sugar and ethanol yield due to physical differences between scales [100, 101], along with other technical and financial issues that may arise.

For now, most of the studies for bioethanol production from coconut husk have focused on the pretreatment and hydrolysis steps; less than half of the articles have addressed the fermentation to ethanol. It is important to include fermentation data because even hydrolysates with high sugar concentration may present problems when put to fermentation due to the presence of inhibitors.

It would be helpful to standardize the way research data are reported in order to facilitate the comparison of different processes and steps, but this is not always possible. In the case of the pretreatment step, one should include the biomass composition before and after pretreatment. As for the hydrolysis, as commented before, stating the glucose concentration in the hydrolysate as well as the total fermentable sugars that can be consumed by the microorganism used would help to determine the effectiveness of the hydrolysis and the fermentation. Stating the ethanol yield based on fermentable sugars also helps to compare the fermentation with other studies and may be useful to alert to a possible problem due to inhibitors. It is always helpful to report the concentration of inhibitors to determine if the simple sugars are being further degraded. Moreover, it is important to report the amount of sugar and ethanol obtained from a given mass of coconut husk so that the efficiency of the process may be determined.

Nowadays, the most economical way to produce 2G bioethanol is the biorefinery scheme, which is important for strengthening and supporting the growing biobased economy.

### 5.1. Biorefinery

The world is entering a new scenario where many countries are taking substantial steps towards a biobased economy. New bioproducts are beginning to replace fossil based products, greenhouse gas emissions are decreasing and innovative policies are emerging to support these changes [102]. To establish the foundation of a biobased economy, the use of biomass resources must be efficient and sustainable. That goal can be achieved by biorefinery systems.

In an energy driven biorefinery system, the biomass is primarily used to produce energy (biofuel, power and/or heat), and other byproducts are upgraded to more added-value products to optimize the economic and ecological performance of the whole production process [103]. Larragoiti-Kuri et al. [104] propose a biorefinery using corn cob as a substrate that produces bioethanol and lactic acid from the cellulose fraction, xylitol and succinic acid from xylose (hemicellulose) and lignosulfonates from lignin. They optimized product distribution by using economic potential, specific energy intensity, and safety indexes as criteria.

**Figure 3:**
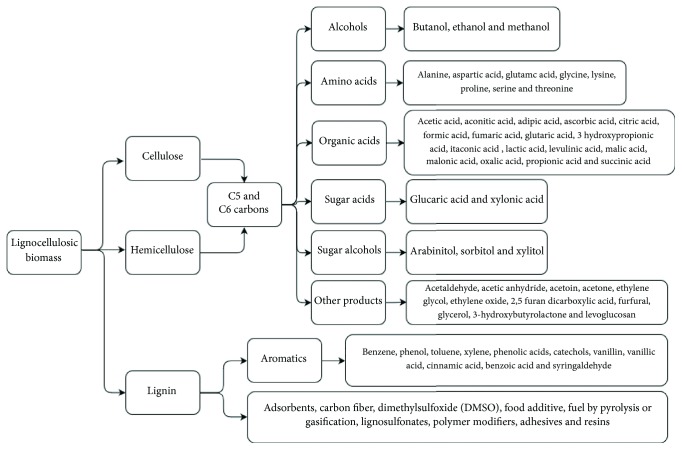
Possible products obtained in a biorefinery [36–38].

Advances in biorefineries allow the development of alternative products to avoid the accumulation of different residues (Figure 3). As an example, 1,3-propanediol obtained from maize residues is important in the formation of polymers. Also, succinic acid removed from various lignocellulosic residues is used in the chemical and pharmaceutical industries. An important alternative to polyethylene is the use of ether amylose derived from various wastes such as sugarcane, potato, and corn [105–108]. From an economic point of view, Gnansounou and Dauriat [95] propose producing a lower diversity of products with stable markets instead of offering a larger number of products, some of which may be unprofitable.

Up to now, all studies of the use of coconut husk for bioethanol production have been made in a small scale and only three studies mention the use of coconut husk as substrate for a biorefinery [30, 32, 33]. These works only discuss the possibility of using byproducts of ethanol production to obtain other substances but no tests to obtain other products have been reported. Gonçalves et al. [33] only suggested the use of sugars, acetic acid, phenolic compounds, and lignin found in the coconut hydrolysate to obtain different products using a biorefinery scheme with no further detail. Later, Gonçalves et al. [30] proposed using their process to make ethanol from coconut husk to obtain also value-added products. They propose that the phenolic compounds obtained during pretreatment and autohydrolysis be used as food additives, since they are antioxidants, while lignin can be used to produce pharmaceutical and veterinarian bioactive compounds and thermoplastic polymers, as well as for energy production through gasification or pyrolysis. They also suggest that xylans obtained from hemicellulose undergo another autohydrolysis to obtain xylooligosaccharide that can be employed in food and pet feed. They suggest that other substances present in the liquors can be used to obtain further products but no specific applications are mentioned. On the other hand, studies not focused on ethanol production show the potential of coconut husks to produce furfural, levulinic acid, formic acid, and acetic acid [109, 110].

Other applications for coconut husks different from ethanol production and possible byproducts were found, such as polymer composites [111, 112] and adsorbents to remove a wide range of water pollutants [113]. As the focus of this work is ethanol production, further studies should be made to see if the biomass remaining after the chosen process to obtain ethanol can still be used for these purposes and analyze if this strategy is economically viable.

## 6. Conclusion

Lignocellulosic ethanol production is a multistep process with many factors that can greatly affect its efficiency. The published coconut husk studies were performed in different conditions throughout the ethanol production process, so it is important to analyze various parameters to define which procedure as a whole has the best results. The final objective is to obtain the highest ethanol concentration per mass of initial substrate for the lowest price, which translates to simpler processes with less energy consumption (lower temperature, pressure, and process time) and less reagents that at the same time have to be low-cost.

Using the concentration of sugars after hydrolysis as a comparison parameter to determine the best method to produce bioethanol is not the best strategy, since studies that use SSF and SSSF do not show the obtained sugars because they are consumed as they are produced. Additionally, most of the works report sugars concentration as total reducing sugars but not all these sugars are fermentable, so estimates of ethanol production may not reflect reality. On the other hand, ethanol yield is related to the transformation of those sugars into ethanol, so comparing these results reflects the ability of the microorganism to ferment the sugars that are present in the hydrolysate. This study showed that alkaline pretreatment is the best method in the case of coconut husk. The highest ethanol yields using coconut husk as a substrate were obtained by Gonçalves et al. [33] using SSSF. The highest ethanol concentration was obtained by Jannah and Asip [28] using an alkaline pretreatment and acid hydrolysis, achieving yields above the 4% (w/w) of ethanol required for an economically feasible distillation.

Finally, the most significant coconut producers are economically developing nations and the industrial residues generated by this culture impose a serious environmental problem. Biorefining this material for the production of ethanol and other molecules with greater added value would enable these countries to create new jobs and boost income.

## Figures and Tables

**Table 1:** Chemical composition of green and mature coconut husk (%).

Substrate	Reference	Cellulose	Hemicellulose	Lignin
Green coconut husk	[27]	39.31	16.15	29.79
	[28]	43.40	19.90	45.80
	[29]	32.80	15.90	n.a.
	[30]	33.23	29.14	25.44

Mature coconut husk	[31]	30.47	25.42	33.15
	[32]	29.58	27.77	31.04
	[33]	32.18	27.81	25.02
	[30]	29.58	27.77	31.04

n.a.: not available or present.

**Table 2:** Comparison of pretreatment methods and inhibitors formed during pretreatment.

**Reference**	**Substrates**	**Type**	**Conditions**	**Reported inhibitors **
[52]	Coconut husk	Microwave-assisted-alkaline	2450 MHz	n.a.

[27]	Young coconut husk	Step 1: NaOH 20-30% (w/v) Step 2: NaOH 25% (w/v)	Step 1: 100°C, 2 and 3 h Step 2: 170°C, 3 h	n.a.

[53]	Coconut husk	Microwave-assisted-alkaline	2450 MHz, 20 min	n.a.
		Autohydrolysis	121°C, 1.043 bar, 15 min	
		H_2_SO_4_ 1% (v/v)	40°C, 150 rpm, 24 h, TS 2%	
		NaOH 5% (w/v)	40°C, 150 rpm, 24 h, TS 2%	

[31]	Green coconut shell, mature coconut fiber, mature coconut shell and cactus	Alkaline hydrogen peroxide (H_2_O_2_ 7.35% (v/v), pH 11.5) followed by alkaline delignification (NaOH 4% (w/v))	H_2_O_2_: 25°C, 1 h NaOH: 100 rpm, 100°C, 1 h	n.a.

[61]	Coconut husk, defatted grape seed and pressed palm fiber	No pretreatment. Direct non-enzymatic hydrolysis with subcritical water		Furfural, HMF, 4-hydroxybenzoic acid and vanillin

[47]	Coconut husk	H_2_SO_4_ 1% (v/v)	121°C, 1 h, TS 7.5%	n.a.

[32]	Green coconut shell, mature coconut fiber, mature coconut shell and cactus	Autohydrolysis	160-200°C, 10-50 min, TS 10%	Acetic acid, furfural and HMF

[28]	Coconut fiber	NaOH 3% (w/v)	121°C, 90 min	n.a.

[46]	Coconut husk	NaOH 2.5 mol·L^−1^	Soaking in NaOH: 30 min Autoclaved: 125°C, 30 min	Phenolic compounds

[49]	Green coconut husks	NaOH 5%	121°C, 40 min, TS 5%	Acetic acid

[33]	Mature coconut fiber	Hydrothermal catalyzed with NaOH	160-200°C, 10-50 min	Phenolic compounds, HMF, furfural and acetic acid

[50]	Green coconut mesocarp	NaOH 1-4% (w/v)	25°C, 200 rpm, 1-24 h, TS 18%	Acetic acid, formic acid, phenolic compounds (various). NO levulinic acid, furfural or HMF detected

[51]	Green coconut husk	Acid-alkaline (H_2_SO_4_ 0.6 mol·L^−1^ and NaOH 4% (w/v))	Acid: 121°C, 15 min, TS 20% Alkaline: 121°C, 30 min	n.a.
		Alkaline hydrogen peroxide (H_2_O_2_ 7.35% (v/v), pH 11.5)	Room temperature, 100 rpm, 1 h, TS 4%	

[54]	Coconut coir fibers	Acidified aqueous glycerol	130°C, 400 rpm, 30 and 60 min, TS 3.3 and 5%	n.a.
		Aqueous glycerol		

[62]	Coconut husk, defatted grape seed, sugarcane bagasse and pressed palm fiber	No pretreatment. Direct non-enzymatic hydrolysis with subcritical water + CO_2_		Furfural, HMF, 4-hydroxybenzoic and vanillin

[48]	Green coconut husk	NaOH 1-2% (w/v)	200 rpm, 25°C, 1 h	Acetic acid, formic acid, phenolic compounds (various) and fatty acids. NO levulinic acid, furfural or HMF detected

[29]	Coconut fiber	NaOH 1 and 2% (w/v) -/+ Tween® 80	121°C, 10-30 min, TS 10%	Acetic acid and phenolic compounds. NO furfural or HMF detected
		H_2_SO_4_ 1.5 and 3% (w/v) -/+ Tween® 80	121°C, 10-60 min, TS 15%	
		Autohydrolysis -/+ Tween ® 80	121°C, 10-60 min, TS 10 and 15%	

[30]	Green coconut shell, mature coconut fiber, mature coconut shell and cactus	NaClO_2_ (0.93% (w/v)) - C_2_H_4_O_2_ (0.31% (v/v)) followed by autohydrolysis	NaClO_2_-C_2_H_4_O_2_: 75°C, 1-4 h, TS 3.1% Autohydrolysis: 200°C, 50 min, TS 10%	Phenolic compounds, HMF, furfural and acetic acid

TS: total solids loadings (w/v); n.a.: not available or not present.

**Table 3:** Comparison of hydrolysis methods and maximum final sugar concentrations.

**Reference**	**Pretreatment**	**Hydrolysis strategy**	**Enzymes**	**Enzyme concentration**	**Conditions**	**Maximum Sugars concentration**
[52]	Microwave-assisted-alkaline	SSF	Cellusclast® 1.5 L and Pectinex® Ultra SP-L	n.a.	30°C, 150 rpm, 96 h, TS 2.5%	n.p.

[27]	2 steps with NaOH	SHF	Celluclast ®1.5L and Novozyme 188 (Novozymes A/S; Denmark)	15 FPU·(g substrate)^−1^ and 15 IU·(g substrate)^−1^	50°C, 140 rpm, 72 h, pH 4.8, TS 5%	22.8 g glucose·L^−1^
		
		SSF			37°C, 72 h, pH 5.5, TS 5%	n.p.

[53]	Microwave-assisted-alkaline	Only hydrolysis	Cellusclast ®1.5 L and Pectinex ® Ultra SP-L	0.5% (v/v) each	35°C, 150 rpm, 5 days, TS 1%	2.8 g TRS ·L^−1^
	Autohydrolysis					Aprox 0.7 g TRS ·L^−1^
	H_2_SO_4_					Aprox 0.7 g TRS ·L^−1^
	NaOH					Aprox 1.4 g TRS ·L^−1^

[31]	Alkaline hydrogen peroxide + alkaline delignification	SSF mature coconut fiber	Cellic®CTec2 and HTec2	30 FPU·(g substrate)^−1^, 75 CBU·(g substrate)^−1^ and 130 IU·(g substrate)^−1^	30°C, 48 h, TS 4%	Aprox 19 g glucose·L^−1^*∗*
		SSSF mature coconut fiber			Prehydrolysis: 50°C, 8 h SSF: 30°C, 40 h TS 4%	

[61]	No pretreatment.	Only hydrolysis	Non-enzymatic hydrolysis with subcritical water			3.4 g monosaccharides ·(100 g substrate)^−1^

[47]	H_2_SO_4_	Only hydrolysis	Celluclast ® and Novozyme 188 (Novozymes A/S; Denmark)	0.33 mL of each	50°C, 150 rpm, 72 h, pH 4.8, TS 0.1-2%	1.2 g TRS ·L^−1^

[32]	Autohydrolysis TS 10%	SSF green coconut shell	Cellic® CTec2 and HTec2	30 FPU·(g substrate)^−1^, 75 CBU·(g substrate)^−1^ and 130 IU·(g substrate)^−1^	30°C, 48 h, TS 4%	Aprox 13 g glucose·L^−1^*∗*
		SSSF green coconut shell			Prehydrolysis: 50°C, 12 h SSF: 30°C, 36 h, TS 4%	

[28]	NaOH	SHF	Non-enzymatic hydrolysis with 1-4% (v/w) H_2_SO_4_, 121°C, 2 h			8.4% (w/v) glucose

[46]	NaOH	SHF	Cellulase® 26921, Novozyme188 (Novozymes A/S; Denmark) and enzymes from coconut husk isolated fungi	7.5 FPU·(g substrate)^−1^	50°C, 96 h, pH 5	50 mM TRS

[49]	NaOH	SHF	Accelerase® 1500	2% (v/v)	50°C, 150 rpm, 72 h. TS 1%	8.7 g TRS ·L^−1^

[33]	Hydrothermal catalyzed with NaOH	SSF	Cellic® CTec2 and HTec2	30 FPU·(g substrate)^−1^, 75 CBU·(g substrate)^−1^ and 130 IU·(g substrate)^−1^	30°C, 48 h, TS 4%	Aprox 16 g glucose·L^−1^*∗*
		SSSF			Pre-hydrolysis: 50°C, 12 h SSF: 30°C, 36 h, TS 4%	

[50]	NaOH	SHF	AlternaFuel® CMAX	3.75, 7.5 and 15 FPU·(g substrate)^−1^	50°C, 200 rpm, 96 h, pH 6, TS 17%	8.7% (w/v) sugars

[51]	Acid-alkaline	Only hydrolysis	Celluclast ®1.5 L	20.0 FPU·(g substrate)^−1^, 20.0 CBU·(g substrate)^−1^ and 10.0 XU·(g substrate)^−1^	50°C, 150 rpm, 72 h, TS 5%	Aprox 9 g TRS ·L^−1^
	Alkaline hydrogen peroxide TS 4%					Aprox 11 g TRS ·L^−1^

[54]	Acidified aqueous glycerol	SSF	Enzyme from *T. reseei*	10 FPU·(g substrate)^−1^	37°C, 120 rpm, 96 h	n.p.
	Aqueous glycerol					

[62]	No pretreatment.	Only hydrolysis	Non-enzymatic hydrolysis with subcritical water + CO_2_			1.7 g monosaccharides ·(100 g substrate)^−1^

[48]	NaOH	SHF	AlternaFuel® CMAX	15 FPU·(g substrate)^−1^ each time	50°C, 200 rpm, 96 h, pH 6, TS 24 and 29%	9.7% (w/v) sugars

[29]	NaOH + Tween® 80 Autohydrolysis	Only hydrolysis	*T. reesei* ATCC 26921, *β*-glucosidases and xylanases	20.0 FPU·(g substrate)^−1^, 20.0 CBU·(g substrate)^−1^ and 10.0 FXU/·(g substrate)^−1^	50°C, 150 rpm, 96 h, TS 5%	0.5 g TRS ·(g substrate)^−1^ 0.1 g TRS ·(g substrate)^−1^

[30]	NaClO_2_- C_2_H_4_O_2_/ autohydrolysis	Only hydrolysis	Cellic® CTec2 and HTec2	10 FPU·(g substrate)^−1^, 30 CBU·(g substrate)^−1^ and 40 IU·(g substrate)^−1^	50°C, 150 rpm, 96 h, TS 4%	Aprox 24 g glucose·L^−1^

n.a.: not available or present; TS: total solids loadings (w/v); n.p.: not present because of SSF or SSSF; *∗* values obtained from liquor after enzymatic hydrolysis but not used for fermentation (SSF and SSSF).

**Table 4:** Comparison of fermentation conditions, ethanol concentration, and yield.

**Reference**	**Pretreatment**	**Fermentation strategy**	**Microorganisms**	**Conditions**	**Maximum Ethanol concentration (g/L or **%**)**	**Ethanol yield (g ethanol/ g sugars or **%**)**
[53]	Microwave-assisted-alkaline	SSF	*S. cerevisiae* ATCC 36858	30°C, 150 rpm, 96 h	0.09% (w/w)	n.a.

[27]	2 steps with NaOH	SHF	*S. cerevisiae*	37°C, 72 h, pH 5.5.	2.28% (w/v)*∗*	Approx 85%
		SSF			1.03% (w/v)	

[31]	Alkaline hydrogen peroxide + alkaline delignification	SSF	*S. cerevisiae*, *P. stipitis* and *Z.mobilis*	30°C, agitation depending on microorganism, 48 h	*S. cerevisiae* 8.44 g·L^−1^ *P.stipitis* 9.12 g·L^−1^ *Z. mobilis* 8.27 g·L^−1^	*S. cerevisiae* 0.43 *P.stipitis* 0.40 *Z. mobilis* 0.42
		SSSF		30°C, agitation depending on microorganism, 40 h	*S. cerevisiae* 9.32 g·L^−1^ *P.stipitis* 10.17 g·L^−1^ *Z. mobilis* 8.91 g·L^−1^	*S. cerevisiae* 0.45 *P.stipitis* 0.43 *Z. mobilis* 0.44

[32]	Autohydrolysis	SSF	*S. cerevisiae*, *P. stipitis* and *Z.mobilis*	30°C, agitation depending on microorganism, 48 h	*S. cerevisiae *7.44 g·L^−1^ *P.stipitis* 8.47 g·L^−1^ *Z. mobilis* 7.30 g·L^−1^	*S. cerevisiae* 0.44 *P.stipitis* 0.43 *Z. mobilis* 0.43
		SSSF		30°C, agitation depending on microorganism, 40 h	*S. cerevisiae* 7.71 g·L^−1^ *P.stipitis* 8.78 g·L^−1^ *Z. mobilis* 7.63 g·L^−1^	*S. cerevisiae* 0.45 *P.stipitis* 0.44 *Z. mobilis* 0.45

[28]	NaOH	SHF	*S. cerevisiae*	150 rpm, 11 days, pH 4.5-5	5.9%	n.a.

[49]	NaOH	SHF	*S. cerevisiae*	30°C, 100 rpm, 9 h	7 g·L^-1 +^	n.a.

[33]	Hydrothermal catalyzed with NaOH	SSF	*S. cerevisiae*, *P. stipitis* and *Z.mobilis*	30°C, agitation depending on microorganism, 48 h	*S. cerevisiae* 10.91 g·L^−1^ *P.stipitis* 10.96 g·L^−1^ *Z. mobilis* 10.81 g·L^−1^	*S. cerevisiae* 0.44 *P.stipitis* 0.45 *Z. mobilis* 0.43
		SSSF		30°C, agitation depending on microorganism, 36 h	*S. cerevisiae* 11.65 g·L^−1^ *P.stipitis* 11.29 g·L^−1^ *Z. mobilis* 11.64 g·L^−1^	*S. cerevisiae* 0. 47 *P.stipit*is 0.46 *Z. mobilis* 0.47

[50]	NaOH	SHF	*S. cerevisiae* strains Ethanol Red and GSE16- T18	35°C, 100 rpm, 103 h, pH 5.5	3.73% (v/v)	0.43

[54]	Acidified aqueous glycerol	SSF	*S. cerevisiae* Hansen 2055	37°C, 150 rpm, 72 h	8.97 g·L^−1^	n.a.
	Aqueous glycerol				2.66 g·L^−1^	n.a.

[48]	NaOH	SHF	*S. cerevisiae* GSE16- T18	35°C, 100 rpm, 72 h, pH 5.5	4.33% (v/v)	0.41

n.a.: not available or not present; *∗* with 50 g·L^−1^ of initial glucose instead of the 22.8 g·L^−1^ reported from the hydrolysis. No explanation for the rise of sugar concentration was found. ^+^ with aprox 16 g·L^−1^ of initial glucose instead of the 8.7 g·L^−1^ reported from the hydrolysis. No explanation for the rise of sugar concentration was found.
